# Transthoracic Ultrasound in Infectious Organizing Pneumonia: A Useful Guide for Percutaneous Needle Biopsy

**DOI:** 10.3389/fmed.2021.708937

**Published:** 2021-07-19

**Authors:** Donato Lacedonia, Carla Maria Irene Quarato, Cristina Borelli, Lucia Dimitri, Paolo Graziano, Maria Pia Foschino Barbaro, Giulia Scioscia, Antonio Mirijello, Michele Maria Maggi, Gaetano Rea, Beatrice Ferragalli, Salvatore De Cosmo, Marco Sperandeo

**Affiliations:** ^1^Institute of Respiratory Diseases, Policlinico Universitario “Riuniti” di Foggia, Foggia, Italy; ^2^Department of Medical and Surgical Sciences, University of Foggia, Foggia, Italy; ^3^Unit of Radiology, Istituto di Ricovero e Cura a Carattere Scientifico (IRCCS) Casa Sollievo della Sofferenza, San Giovanni Rotondo, Italy; ^4^Unit of Patology, Istituto di Ricovero e Cura a Carattere Scientifico (IRCCS) Casa Sollievo della Sofferenza, San Giovanni Rotondo, Italy; ^5^Department of Internal of Medicine, Istituto di Ricovero e Cura a Carattere Scientifico (IRCCS) Fondazione Casa Sollievo della Sofferenza, San Giovanni Rotondo, Italy; ^6^Department of Emergency Medicine, Istituto di Ricovero e Cura a Carattere Scientifico (IRCCS) Fondazione Casa Sollievo Della Sofferenza, San Giovanni Rotondo, Italy; ^7^Department of Radiology, “Vincenzo Monaldi” Hospital—Association of periOperative Registered Nurses (AORN) Ospedale Dei Colli, Naples, Italy; ^8^Department of Radiology, “SS. Annunziata” Hospital, University of Chieti, Chieti, Italy; ^9^Unit of Interventional and Diagnostic Ultrasound of Internal Medicine, Istituto di Ricovero e Cura a Carattere Scientifico (IRCCS) Fondazione Casa Sollievo della Sofferenza, San Giovanni Rotondo, Italy

**Keywords:** lung ultrasound, chest computed tomography, chronic pneumonia, organizing pneumonia, diagnostic accuracy, lung ultrasound-guided percutaneous needle biopsy

## Abstract

In patients presenting with classical features of CAP (i.e., new peripheral pulmonary consolidations and symptoms including fever, cough, and dyspnea), a clinical response to the appropriate therapy occurs in few days. When clinical improvement has not occurred and chest imaging findings are unchanged or worse, a more aggressive approach is needed in order to exclude other non-infective lesions (including neoplasms). International guidelines do not currently recommend the use of transthoracic ultrasound (TUS) as an alternative to chest X-ray (CXR) or chest computed tomography (CT) scan for the diagnosis of CAP. However, a fundamental role for TUS has been established as a guide for percutaneous needle biopsy (US-PNB) in pleural and subpleural lesions. In this retrospective study, we included 36 consecutive patients whose final diagnosis, made by a US-guided percutaneous needle biopsy (US-PTNB), was infectious organizing pneumonia (OP). Infective etiology was confirmed by additional information from microbiological and cultural studies or with a clinical follow-up of 6–12 months after a second-line antibiotic therapy plus corticosteroids. All patients have been subjected to a chest CT and a systematic TUS examination before biopsy. This gave us the opportunity to explore TUS performance in assessing CT findings of infective OP. TUS sensitivity and specificity in detecting air bronchogram and necrotic areas were far lower than those of CT scan. Conversely, TUS showed superiority in the detection of pleural effusion. Although ultrasound findings did not allow the characterization of chronic subpleural lesions, TUS confirmed to be a valid diagnostic aid for guiding percutaneous needle biopsy of subpleural consolidations.

## Introduction

Patients presenting to the Emergency Department (ED) with respiratory symptoms, such as cough, purulent sputum, and dyspnea, may show pulmonary consolidations on standard chest x-ray (CXR). The most common cause for new-onset pulmonary consolidations is an infective pneumonia (1, 2). However, in a patient with chronic symptoms, persistent consolidations, not reducing in size or even worsening on follow-up CXR, open the scenario for a completely different spectrum of differential diagnoses, including inadequately treated or atypical infections, lung abscess, organizing pneumonia (OP), malignancy, chronic eosinophilic pneumonia, sarcoidosis, or vasculitis (3, 4).

The exact incidence and prevalence of OP are unknown. One study estimated the incidence of secondary OP at 0.87/100,000/year and cryptogenic OP at 1.10/100,000/year (5), thus configuring a relatively rare condition. Infection is an increasing cause of OP (6), probably due to the rising number of antibiotic-resistant bacteria and our decreasing capacity to eradicate them (7). Plugs of granulation tissue consisting of a mixture of chronic inflammatory cells and fibroblasts embedded in a myxoid matrix filling the distal airspaces constitute the histological hallmark of OP (8). Organization is believed to be a consequence of a prolonged inflammatory reaction causing alveolar epithelial injury with cell necrosis, denudation of the basal laminae and intra-alveolar fibrinous exudate (9).

In case of persistent consolidations, performing a chest computed tomography (CT) scan is helpful, as it can provide clues to narrow the differential diagnosis and further delineate the distribution and extent of disease (1, 4). Chest CT findings of OP include focal or multiple consolidations mixed with patchy ground glass opacities mainly concentrated in the subpleural region (6). Misinterpretation as malignancy is common. Moreover, infection may also be associated with neoplastic lesions, which should therefore be excluded.

Despite the fact that international guidelines do not currently recommend the use of transthoracic ultrasound (TUS) for the diagnosis of CAP, this imaging method can detect peripheral pulmonary nodules or masses when they are adherent to the parietal pleura (10, 11). So, in case of non-resolving subpleural consolidations, ultrasound-guided percutaneous needle biopsy (US-PTNB) is an appealing alternative to surgical biopsy for histological assessment, as it is less invasive and associated with fewer complications (12–14). Otherwise, an objective evaluation of TUS as an accurate imaging method for the morphological characterization of persistent lung consolidations is still lacking and debated (15).

In this retrospective brief report, we have included 36 patients whose final diagnosis after US-PTNB was infectious OP. All the patients performed a pre-operative chest CT scan and an accurate TUS examination before proceeding with the biopsy procedure. This gave us the opportunity to compare the diagnostic performance of TUS with respect to chest CT (gold standard) in the identification of findings suggestive for OP and to assess the effectiveness and safety of US-PTNB for the biopsy of subpleural lung lesions.

## Materials and Methods

The medical records of 36 patients diagnosed with infectious OP from January 2015 to November 2019 in our Research Institute “Fondazione Casa Sollievo della Sofferenza Hospital” (San Giovanni Rotondo, Italy) were reviewed. All the patients received a previous attempt at broad-spectrum antibiotic therapy (of at least 10–14 days duration), despite which they showed persistence of subpleural consolidation on routine follow-up imaging. As an appropriate assessment was considered urgently needed to guide appropriate therapy, these patients have been scheduled for histological assessment by US-guided percutaneous needle biopsy (US-PTNB) in our Unit of Interventional and Diagnostic Ultrasound of Internal Medicine. All the patients were subjected to a pre-operative chest CT scan and a concurrent TUS examination. The decision to biopsy was made after the exclusion of contraindications, such as bleeding diatheses (i.e., PT-INR > 1.5 or platelet count < 30,000), severe pulmonary emphysema, severe pulmonary hypertension (i.e., pulmonary artery pressure >90 mmHg), recent myocardial infarction, or unstable angina. Infective etiology was confirmed by additional information from microbiological and cultural studies on blood, sputum, and bronchoalveolar lavage obtained with bronchoscopic examination at admission or by the reliever of symptoms and the reduction/disappearance of consolidations at 6–12 months of clinical–radiological follow-up from starting a second-line antibiotic therapy plus an appropriate corticosteroid treatment.

The primary endpoint was to assess the performance of TUS vs. chest CT (gold standard) in the identification of findings suggestive for infectious OP. The secondary endpoint was to assess the effectiveness and safety of TUS in guiding percutaneous needle biopsy of subpleural lung consolidations for histological assessment.

All the procedures were performed in accordance to the amended Declaration of Helsinki and the local institutional review board approved the protocol.

### Pre-operative Chest CT

Pre-operative enhanced CT examinations were performed using a multi-detector CT scanner with 64 channels. The detailed parameters for CT acquisition were as follows: tube voltage, 120 kVp; tube current, standard (reference mAs, 60–120); slice thickness, 0.5 mm; reconstruction interval, 0.5–1.0 mm. CT images were acquired at full inspiration with the patient in the supine position.

According to the Fleischner Society's glossary of terms for thoracic imaging (8), a consolidation was defined as “…a homogeneous increase in pulmonary parenchymal attenuation that obscures the margins of vessels and airway walls” and ground glass opacities were defined as areas of “…hazy increased opacity of lung with preservation of bronchial and vascular margins”. A lung consolidation has been considered as “persistent” if it lasted for more than a month since first detection and an adequate empirical antibiotic therapy (of at least 10–14 days duration).

CT scans were reviewed by two expert radiologists to reach consensus. The following characteristics were recorded for each lesion: size and location; presence/absence of air bronchogram, defined as a pattern of air-filled (low-attenuation) bronchi on a background of opaque (high-attenuation) airless lung (8); presence/absence of necrosis, defined as distinct areas of low attenuation on CT scan; and presence/absence of additional pleural effusion.

### TUS Examination

For TUS examination, we employed an Esaote MyLab-9 scanner (Esaote-Biomedica, Genoa, Italy) and a convex multi-frequency probe (2–8 MHz). The following machine setting was used: depth varying between 70 and 140, time gain compensation (TGC) of no more than 50%, focus pointed at the hyperechoic pleural line, and activation of the tissue harmonic imaging. Patients were examined in a sitting or semi-sitting position. Each hemithorax was systematically explored, from the lung base to the apex, posteriorly (along the para-vertebral, hemi-scapular, and posterior-axillary lines), laterally (along the middle-axillary line), and anteriorly (along anterior-axillary, hemi-clavicular, and para-sternal lines) with intercostal longitudinal and transversal scans. Recorded videoclips for each subject were reviewed by three expert sonographers who were blinded to concurrent CT scan results. Pulmonary consolidations were categorized according to their morphology as follows: size; ultrasound pattern, classified as “hypoechoic” or “mixed” (i.e., hyper/hypoechoic); regular/irregular shape; presence/absence of the sonographic “air bronchogram,” defined as hyperechoic linear or lenticular spots inside a consolidation; presence/absence of “necrosis,” identified as focal anechoic areas within a consolidation; and presence/absence of pleural effusion.

### US-PTNB Procedure

US-PTNB was performed by an expert sonographer with 32 years of experience in interventional ultrasound with the “modified Menghini” technique (14). A semi-automatic 18-gauge Menghini-type needle and a dedicated multifrequency convex transducer (3.5–8 MHz) with a central opening for needle insertion were employed. Once the lesion was well-framed on standard US B-mode, the needle was inserted in the probe's path and guided within the lesion in real time. Subsequently, the charged syringe plunger was released, removing the stylet and applying suction. The operator made “a back and forth” movement with the needle in order to facilitate the sample of pathological material. The patient was instructed to hold the breath during the procedure.

Patients were also closely monitored for 3–4 h after the procedure. Expiratory chest X-rays were performed to rule out an iatrogenic pneumothorax.

Histological diagnoses were made by a pathologist with over 20 years of experience in lung pathology. Biopsy was considered “diagnostic” if it acquired an adequate volume of pathological material that yielded a definitive histological diagnosis. Biopsies that showed only necrosis, fibrous tissue, or normal bronchial tissue were considered “inadequate.”

### Statistical Analysis

Data were expressed as means ± standard deviation (SD) for continuous variables and as absolute numbers and frequencies (*n*, %) for nominal data. Comparison between lesion mean size on chest CT and TUS was analyzed by the paired Student's *t*-test. A *p*-value of <0.05 was considered statistically significant. Chest CT was regarded as the “gold standard” method for correct assessment of findings of chronic pneumonia. Concordant and discordant results between chest CT and TUS were analyzed with a 2 × 2 correlation matrix. Agreement was quantified by Cohen's *k* coefficients, with *k* values from 0.81 to 1.00 indicating almost perfect agreement; 0.61 to 0.80, substantial agreement; 0.41 to 0.60, moderate agreement; 0.21 to 0.40, fair agreement; 0.01 to 0.20 slight agreement; and <0, no agreement. TUS sensitivity, specificity, positive and negative predictive values, and positive and negative likelihood ratios in detecting findings of chronic pneumonia were calculated with a 95% confident interval (CI). The empiric receiver operating characteristic (ROC) curve analysis was used to study the diagnostic performance of LUS vs. chest CT in discriminating findings of infective OP. We defined area under the ROC curve (AUC) values of 0.50–0.59, 0.60–0.69, 0.70–0.79, and ≥0.80 as none, poor, acceptable, and excellent discrimination, respectively.

## Results

Between January 2015 and November 2019, 36 patients, 30 males (83%) and 6 females (17%), underwent US-PTNB for the assessment of persistent subpleural lung consolidations and received an histological diagnosis of OP. The mean age was 47 ± 15 years (range 23–77) and 56% of patients were smokers. Markers of systemic inflammation at admission were elevated (mean WBC count: 14.58 ± 2.55; mean CRP: 86 ± 30; mean PCT: 0.17 ± 0.08).

All the patients (100%) showed multiple lung consolidations on chest CT. The consolidation that was most clearly viewable on TUS examination was selected as target for systematic TUS study and subsequent US-PTNB (Table 1).

**Table 1:** Characteristics of the biopsied lesions on CT scan and TUS examination.

**Lesions (*n* = 36)**	
**CT results**	
**Diameter, cm**	
Mean ± SD	4.15 ± 0.93
Min–max	1.75–6.75
**CT findings (*****n*****, %)**	
Air bronchogram	15 (42%)
Necrosis	11 (31%)
Pleural effusion	13 (36%)
**TUS results**	
**Diameter, cm**	
Mean ± SD	3.92 ± 0.88
Min–max	1.75–6.50
**Pattern (*****n*****, %)**	
Hypoechoic	20 (56%)
Mixed (hyper/hypoechoic)	16 (44%)
**Shape (*****n*****, %)**	
Irregular	15 (42%)
Regular	21 (58%)
**TUS findings (*****n*****, %)**	
Hyperechoic spots/striae	22 (61%)
Anechoic areas	19 (53%)
Pleural effusion	18 (50%)

We did not find a statistically significant difference between the mean diameter of the lesions measured on chest CT scan and that measured on TUS examination, although lesions appeared slightly smaller on TUS (4.15 ± 0.93 vs. 3.92 ± 0.88, *p* = 0.3).

On TUS examination, a hypoechoic or mixed hyper/hypoechoic pattern (56 vs. 44%) and an irregular or regular shape (42 vs. 58%) represented equally frequent characteristics.

**Figure 1:**
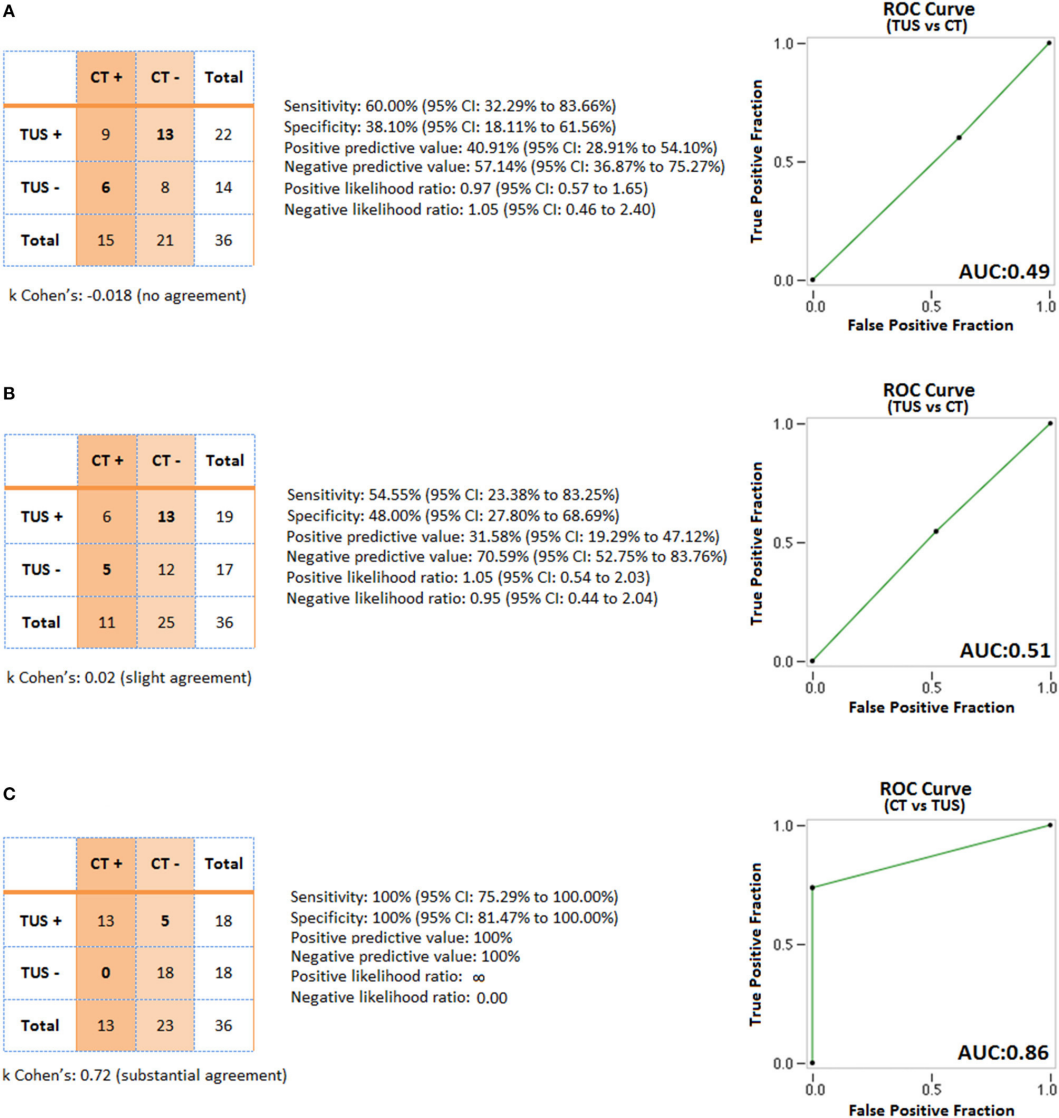
2 × 2 correlation matrixes of concordance and discordance between chest CT and TUS and ROC curves for the following signs of chronic pneumonia: **(A)** Air bronchogram; **(B)** Necrosis; **(C)** Pleural effusion.

Presence of air bronchogram was detectable on chest CT scan in 15/36 (42%) lesions. Inner hyperechoic striae or spot on TUS examination were assessed in 22/36 (61%) lesions. The “sonographic air bronchogram” matched with the actual presence of air bronchogram on chest CT scan in 9 cases. Otherwise, in 13 cases, TUS examination assessed the presence of hyperechoic striae within the consolidation, but the corresponding chest CT scan was negative for the presence of air bronchogram (TUS “false positives”), and in 6 cases, the chest CT scan assessed the presence of air bronchogram but the corresponding TUS examination was negative. Cohen's *k* coefficient assessed no agreement between the two diagnostic tests (*k* = −0.018). TUS showed a sensitivity of 60.00% (95% CI: 32.29 to 83.66%), a specificity of 38.10% (95% CI: 18.11 to 61.56%), a positive predictive value of 40.91% (95% CI: 28.91 to 54.10%), a negative predictive value of 57.14% (95% CI: 36.87 to 75.27%), a positive likelihood ratio of 0.97 (95% CI: 0.57 to 1.65), and a negative likelihood ratio of 1.05 (95% CI: 0.46 to 2.40) in detecting air bronchogram. An AUC value of 0.49 highlighted no discrimination (Figure 1A).

Necrosis was assessed in 11/36 (31%) lesions on chest CT scan. The discovery of anechoic regions within the consolidation on TUS occurred in 19/36 (53%) lesions. The finding of anechoic areas within the consolidation on TUS matched with the evidence of necrosis on chest CT in six cases. On the contrary, there were 13 lesions presenting anechoic regions on TUS examination in which presence of necrosis was not confirmed by the corresponding CT scan (TUS “false positives”) and five cases where CT scan allowed identification of necrotic areas but the corresponding TUS examination was falsely negative. According to Cohen's *k* coefficient, there were slight agreement between the two diagnostic tests (*k* = 0.02). TUS showed a sensitivity of 54.55% (95% CI: 23.38 to 83.25%) and a specificity of 48.00% (95% CI: 27.80 to 68.69%) in assessing necrosis. TUS positive and negative predictive values were 31.58% (95% CI: 19.29 to 47.12%) and 70.59% (95% CI: 52.75 to 83.76%), respectively. Positive and negative likelihood ratios were 1.05 (95% CI: 0.54 to 2.03) and 0.95 (95% CI: 0.44 to 2.04), respectively. An AUC value of 0.51 highlighted no discrimination (Figure 1B).

A quantity of para-pneumonic fluid was detectable in 13/36 (36%) patients at chest CT scan and in 18/36 (50%) patients at ultrasound examination. TUS examination allowed the detection of pleural effusion in all cases judged positive on chest CT. Furthermore, TUS assessed the presence of mild pleural effusion in five other cases not identified on chest CT scan. According to Cohen's *k* coefficient, there were substantial agreement between the two diagnostic tests (*k* = 0.72). However, TUS showed superiority in the detection of pleural effusion compared to chest CT. In particular, TUS showed a sensitivity of 100% (95% CI: 75.29 to 100.00%) and a specificity of 100% (95% CI: 81.47 to 100.00%) for pleural effusion; positive and negative predictive values were both 100%. An AUC value of 0.86 highlighted an excellent discrimination of chest CT vs. TUS for pleural effusion (Figure 1C).

US-PTNB resulted in an adequate volume of pathologic material allowing a definitive histological diagnosis of OP in all the cases. The mean number of needle passes per biopsy was 1.07 ± 0.14. Macroscopically inadequate sampling for which was required the immediate repetition of the biopsy procedure during the same session occurred in two (5%) cases. The repetition of the biopsy in the same session was decided by the operator using visual inspection in judging the adequacy of the sample obtained. There were no major complications resulting from US-PTNB. Only one small post-biopsy pneumothorax, not requiring placement of a chest tube, occurred over the 36 procedures.

Delayed results from appropriate cultures on bronchoalveolar lavage revealed an infection from mycobacterium tuberculosis in two sputum-negative patients. In addition, in 11 patients, BAL cultures allowed the diagnosis of an infection from multi-drug-resistant bacteria, among which *Streptococcus pneumoniae* (five cases), Enterobacteriaceae (three cases), *Pseudomonas aeruginosa* (one case), *Acinetobacter baumannii* (one case), and *Klebsiella* spp. (one case). In two patients, infection from *P. aeruginosa* and *A. baumannii* was confirmed also by positive blood cultures. In the remaining 23 cases (64%), bronchoscopy with microbiological evaluation was non-diagnostic (8 patients) or has not been carried out (15 patients). A diagnosis of infectious OP was confirmed with clinical–radiological follow-up evaluation at 6 and 12 months after starting a second-line antibiotic therapy plus an appropriate corticosteroid treatment.

## Discussion

Infection is probably a frequent, but underestimated cause of OP. Indeed, proving a causal link between respiratory infections and subsequent organization may be challenging. In the 36 patients included in this brief report, a diagnosis of respiratory infection was suggested by clinical history and elevated values of systemic inflammatory markers (WBC count, CRP, and PCT) at admission (16). However, most of the cases were confirmed by the reduction/disappearance of consolidations at clinical–radiological follow-up after starting a second-line antibiotic therapy plus corticosteroids. Only in a minority of cases was an infection diagnosed from cultures on bronchoalveolar lavage obtained with bronchoscopy.

In recent years, the complementary use of TUS imaging is attracting clinical interest for the study of several pleuropulmonary diseases, including pulmonary edema, pneumothorax, lung fibrosis, and pleural and subpleural lesions (17–20). However, ultrasound images are strongly influenced by the presence of air in the lungs. More than 95% of the ultrasound beam is reflected at the interface between the chest wall tissues and the pleural surface and normal pleuropulmonary interface is visualized as a hyperechoic line followed by reverberation artifacts (i.e., the so-called “A-lines”), moving synchronously with the breaths in real-time examination (i.e., the “gliding sign”) (20). TUS cannot visualize foci of pneumonia, which are not adherent to the pleural surface or are positioned where ultrasound cannot penetrate (e.g., facing the mediastinal pleura or located below the bony structures of the rib cage) (10, 11). Otherwise, when pleural effusions and condensate lung overlook the parietal pleura, facilitation of the ultrasound beam can allow detection of deeper lesions. Most cases of CAP (~80% of cases), as well as OP consolidations, are subpleural, thus most often examinable by TUS (10, 11). Generally, the consolidation size appears smaller at US than on other radiological imaging (i.e., CXR or chest CT) (15). This evidence was confirmed also in our case report, although the difference was not statistically significant. The reason for this result lies in the fact that the periphery of the pneumonia is more air-filled, which results in more artifacts, thus limiting complete visualization of the extent of consolidation (21, 22).

According to literature, inflamed subpleural lung tissue appears on TUS as a mixed hyper/hypoechoic (i.e., “hepatized”) or hypoechoic consolidation of varying size and shape, often showing irregular and blurred deeper margins, as a result of continuity with aerated lung (21, 22). Experts emphasize not only that pneumonia of an atypical etiology may present a different sonomorphology than the one described above but also that the aspect of typical inflammatory lesions may overlap with those caused by less common pathogens (23). The sonographic pattern of persistent subpleural consolidations examined in this case series seems to quite correspond to that described for common CAP, thus confirming that the lung ultrasound does not allow an etiological diagnosis of pneumonia. In adjunct, it should be stressed that, although pneumonia is the most common cause of lung consolidation, other conditions can result in consolidation that appears similar to that of pneumonia at TUS, including cancer (17, 20).

Some authors have stated that in patients with ultrasound-visible alveolar consolidation, the finding of the so-called “dynamic air bronchogram” had a 94% specificity and a 97% positive predictive value for diagnosing pneumonia (24). Sonographic “air bronchograms” have been described as hyperechoic spots or stripes within a consolidation that represent the patent bronchial tree contrasting to the fluid-filled alveoli. The genesis of this ultrasound finding has been attributed to a change in acoustic impedance between consolidated lung and air-filled bronchi. When these structures are observed to propagate distally and proximally with inspiration and expiration, they are defined as “dynamic air bronchograms.” However, no study or meta-analysis so far demonstrated that such lenticular or arborescent hyperechoic images on TUS do really correspond to the CT imaging finding of air bronchogram (25). As a matter of fact, in our study, Cohen's *k* assessed no agreement between CT and TUS in assessing this finding. TUS was unable to detect the air bronchogram in 6/15 (40%) cases. This result is easily explained by the fact that TUS is a 2D imaging method, unlike CT, which allows a 3D study of consolidations. Therefore, some portions of the bronchial tree may not be identified depending on the plane in which the consolidation is cut by the US beam or also because they are located in areas not reachable by US (e.g., behind the bone structures of the thoracic cage). Otherwise, TUS resulted falsely positive for the presence of air bronchogram in 13/29 (62%) cases. Another possible explanation for the presence of hyperechoic striae/spots within a consolidation may be a change in acoustic impedance due to the interposition of also few microns of air between different areas of the studied lesion, deriving from an incomplete contact with the parietal pleura or micro-areas of colliquative necrosis (26). Moreover, the presence of plugs of connective tissue in the airspaces and distal airways, which represents a histological hallmark of OP, makes the distinction between air bronchogram and hyperechoic striae/spots of another nature more difficult. Anyhow, even in lung carcinomas, it is possible to see areas of CT air bronchogram and/or hyperechoic spots and striae at TUS, confirming that this finding cannot be considered as a reliable marker of benign consolidation (27).

TUS B-Mode grayscale showed a low sensitivity and specificity in assessing necrotic areas within consolidations, with only a slight agreement between TUS and chest CT. This evidence has been confirmed by a substantial number of other studies (26, 28–30). Once again, false negatives can be justified by the position occupied by the areas of necrosis within the consolidation and by the physical limitations encountered by ultrasound in the study of lung lesions (26). On the other hand, false positives are related to the heterogeneity of the sonographic pattern shown by lung lesions (21, 22). Some authors have indicated the possibility of distinguishing necrotic areas from other unspecific anechoic areas within a consolidation through the Doppler study of the vascular pattern (31). However, this parameter may not be considered a reliable sign of distinction, because of the presence of “flash artifacts” (i.e., a burst of color signal caused by motion of transducer or the patients' breathing) in most patients (32). For this reason, in the present study, we did not employ Doppler study to assess necrosis.

Inflammatory lung consolidations are frequently associated with basal pleural effusions (21, 22). TUS examination showed a 100% sensitivity in evidencing pleural effusions, compared to chest CT. In addition, in five patients included in our case series, the pleural effusion was so minimal that it was not possible to previously assess it on CT scan (probably because it was layered in supine position). In particular, consolidations placed at the lung base (near the costo-diaphragmatic sinus) accumulated by gravity a little effusion over the lesion that was therefore detectable only during ultrasound examination in a sitting position. Furthermore, the aspect of pleural effusion on TUS can suggest the nature of the fluid, although a definitive diagnosis requires a thoracentesis in order to perform physical, chemical, and microbiological studies. Pleural effusion on ultrasound can appear as anechoic (black), complex non-septated (black with white strands), complex septated (black with white septa), or homogeneously echogenic (white) (33). In general, an anechogenic effusion suggests a transudate, a homogeneous echogenic effusion suggests corpuscular fluids (i.e., hemorrhage or empyema), and the presence of a complex pleural effusion suggests an exudate.

**Figure 2:**
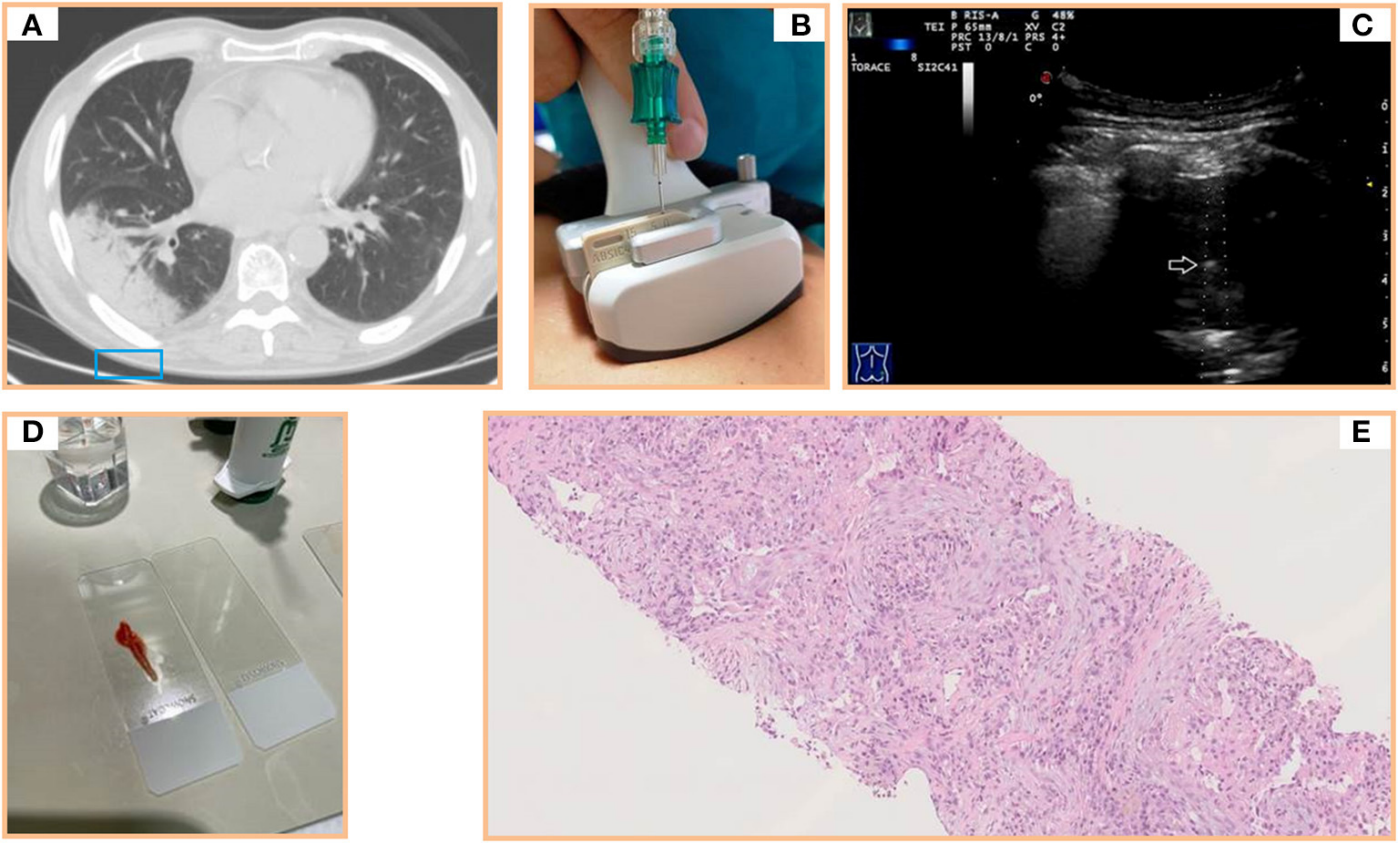
**(A)** Axial chest computed tomography (CT) showing a subpleural pulmonary lesion with inner air bronchograms in the lower right lobe. **(B)** Dedicated ultrasound convex transducers with a central hole for needle set insertion during US-guided biopsy procedure. **(C)** Transthoracic ultrasound scan (TUS) using the dedicated convex probe (3.5–8 MHz) during US-guided biopsy (corresponding to the blue box in A) allowing real-time visualization of the needle (white arrow) in a hypoechoic subpleural lung lesion. **(D)** Specimen suitable for histological and cytological diagnosis. **(E)** Histological examination of a sample from the lesion (hematoxylin and eosin) revealing a mixture of inflammatory cells and fibroblastic plugs within airspaces.

**Figure 3:**
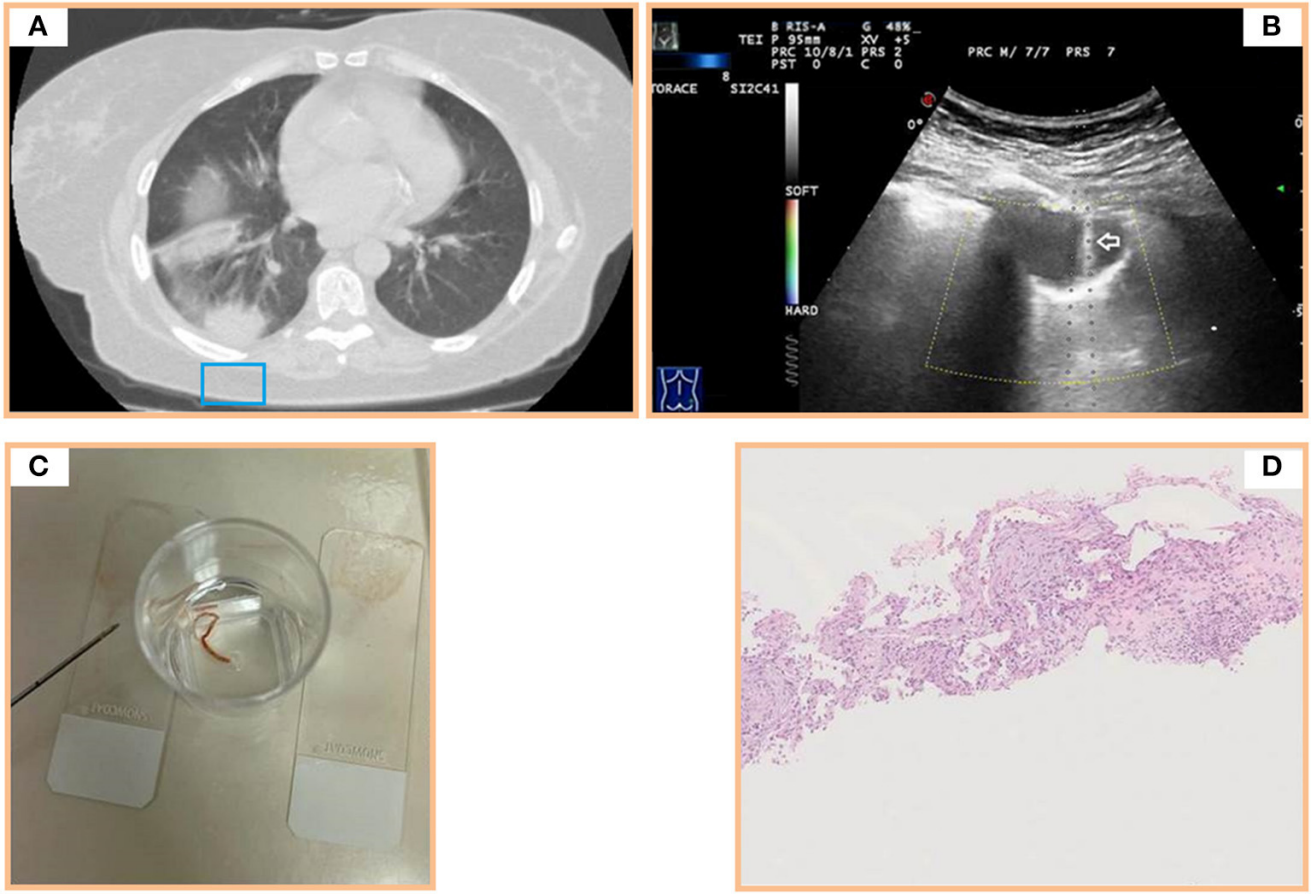
**(A)** Axial chest computed tomography (CT) of multiple subpleural pulmonary consolidations in the lower right lobe. **(B)** Transthoracic ultrasound scan (TUS) using the dedicated convex probe (3.5–8 MHz) during US-guided biopsy (corresponding to the blue box in A) allowing real-time visualization of the needle (white arrow) in the posterior hypoechoic subpleural lung lesion. **(C)** Specimen suitable for histologic and cytologic diagnosis. **(D)** Histological examination of a sample from the posterior subpleural consolidation (hematoxylin and eosin) showing thickened alveolar septa with inflammatory infiltrate and fibroblastic plugs in alveolar sacs.

Briefly, TUS examination is not the ideal imaging method for characterization of subpleural persistent consolidations. The final diagnosis requires confirmatory histological studies on tissue samples. To this regard, our experience confirmed that TUS is a safe and effective method for guiding PNTB of subpleural consolidations. In our study, a diagnosis of OP was possible in all the cases and no major complications followed the biopsy procedure. These excellent outcomes may be related to the skills of the operator and to the type of device used. The use of probes that have a central hole through which the needle set is introduced optimized the procedure, as it allowed to follow the needle in its road in real time, with an image exactly on the line of the target lesion and the transducer (34, 35) (Figures 2, 3). The operator's experience made him able to choose when discarding any sample for which it would not be expected an accurate diagnostic result (e.g., in case of macroscopic areas of necrosis or samples that were too small and/or excessively fragmented) and to immediately repeat the biopsy procedure in the same single session. Furthermore, each lesion in our study was carefully studied on pre-operative CT scan before proceeding with the guided procedure. This allowed us to make an *a priori* evaluation on where to bite the lesion in order to avoid necrosis and to sample viable tissue. Finally, the use of an atraumatic 18-gauge needle allowed us to minimize the occurrence of complications, which appear to be more frequent with needles of a higher caliber (i.e., 14–16 gauge) (14, 35).

Our study finds its main limitations in the retrospective design and in the fact that an ideal sample size, which is one of the requirements for adhering to the STARD guidelines (36), was not pre-established. The retrospective design leaves the possibility of residual confounding and data came from a relatively small number of patients. However, as OP is a relatively rare disease, a retrospective design and small sample sizes characterized also other works on this pathologic condition (6, 16, 37–39). Considering that this study was conducted in one hospital and covered a relatively short period of time, we collected a number of patients that are perfectly in line with the available literature on the topic. Despite these limitations, we believe that the findings of this study may offer useful information on the sonographic appearance of OP and on TUS potentiality in guiding percutaneous biopsy for the histological assessment of this rare condition.

## Conclusion

From this brief report, we can conclude that infectious OP should be considered in cases of persistent infiltrates despite antibiotic treatment. TUS findings in persistent lung consolidations are clearly unspecific and do not allow one to uniquely characterize lesions. In such cases, a CT scan (especially with high-resolution technique) represents certainly the gold standard, as it can delineate the distribution and extent of disease, provide clues to narrow the differential diagnosis, and aid in guidance for further invasive diagnostic procedures, such as bronchoscopy or surgical biopsy. Anyhow, when subpleural consolidations can be detected on TUS examination, this imaging method can safely and effectively guide a percutaneous needle biopsy for histological assessment.

## Data Availability Statement

The raw data supporting the conclusions of this article will be made available by the authors, without undue reservation.

## Ethics Statement

The studies involving human participants were reviewed and approved by Institutional review board of Research Institute “Fondazione Casa Sollievo della Sofferenza Hospital” (San Giovanni Rotondo, Italy). The patients/participants provided their written informed consent to participate in this study.

## Author Contributions

CMIQ and MS contributed to the conception and design of the study and to the acquisition and interpretation of data, and wrote the manuscript and revised it critically. CB, LD, DL, MF, GS, PG, AM, MM, GR, BF, and SD contributed to acquisition and interpretation of data. All authors contributed to manuscript revision and read and approved the submitted version.

## Conflict of Interest

The authors declare that the research was conducted in the absence of any commercial or financial relationships that could be construed as a potential conflict of interest.
